# Reporting of notifiable medical conditions among emergency department doctors in Gauteng

**DOI:** 10.4102/sajid.v41i1.832

**Published:** 2026-07-20

**Authors:** Kate I. Smith, Patricia M. Saffy, Margaret P. Fitchett

**Affiliations:** 1 Division of Emergency Medicine, Department of Family Medicine and Primary Care, School of Clinical Medicine, Faculty of Health Science, University of the Witwatersrand, Johannesburg, South Africa

**Keywords:** notifiable medical conditions, emergency department, notifiable disease, disease reporting, digital disease notification, disease surveillance

## Abstract

**Background:**

Surveillance of notifiable medical conditions (NMCs) is vital for a prompt, appropriate response. In South Africa, studies have demonstrated under-reporting of NMCs despite it being mandatory. Emergency departments (EDs) are often the first point of contact for patients with NMCs.

**Objectives:**

This study aimed to assess ED clinicians’ knowledge, attitudes, and practices regarding the reporting of conditions that are relevant to the ED, and the use of the new mobile application for reporting.

**Method:**

A cross-sectional survey was conducted between November 2024 and April 2025, including 99 doctors at three public hospital EDs in Gauteng. The study assessed knowledge, attitudes and practices regarding NMCs and the use of the recently launched National Institute for Communicable Diseases (NICD) mobile application.

**Results:**

The overall mean knowledge score was 61% (standard deviation [s.d.] 11.2). Knowledge accuracy of category 1 NMCs (mean 72%) exceeded that of category 2 NMCs (mean 56%; *p* < 0.001). Although 95% of respondents were aware that reporting NMCs is mandatory, 49% acknowledged under-reporting. Barriers to reporting included high workload, forgetfulness, and difficulty in locating the paper notification booklet. While 81% of respondents knew about the NICD mobile application, 68% had used it, and 17% had received formal training. Preference for the application versus the paper-based system was expressed by 78% of respondents.

**Conclusion:**

Under-reporting persists primarily because of high workload. A significant knowledge gap exists, particularly concerning category 2 NMCs. While the mobile application was preferred, a lack of formal training hinders its adoption. Staff training, feedback and reminders would improve the timely notification of these conditions.

**Contribution:**

This study identifies under-reporting of NMCs and offers recommendations to strengthen the disease surveillance system and encourages the use of digital technology for reporting.

## Introduction

In South Africa (SA), it is a legal requirement for a healthcare worker to report a notifiable medical condition (NMC), which includes mostly infectious diseases and some non-infectious conditions.^1^ Infectious diseases may spread rapidly, may cause severe disease and vaccination may prevent or limit the spread of some infections. The surveillance of these diseases is important to be able to detect and respond to outbreaks early and to prevent these diseases from becoming a threat to the public.^1^ Data obtained from these surveillance systems inform vaccine development and rapid rollout during outbreaks. The recent COVID-19 pandemic and Ebola outbreaks are examples that have highlighted the global importance of these surveillance systems.^2^

Certain non-infectious conditions, such as pesticide poisoning, maternal death, mercury poisoning or lead poisoning, place a significant burden on healthcare systems. Without accurate reporting of these conditions, the extent of their effect can be underestimated, leading to suboptimal policies to reduce their burden on the population.^3^

Surveillance systems require epidemiological data collection and analysis to discover and respond to health threats that may put the public at risk.^4^ In addition, these systems provide data for research and inform decisions about resource allocation in an effort to prevent these conditions or identify gaps in current prevention programmes.^5^ Surveillance systems include clinician reporting of notifiable conditions as well as laboratory surveillance.^6,7,8^

There are four categories under which NMCs are grouped, based on the urgency of notification.^1^ Category 1 includes conditions such as malaria, measles, meningococcal disease and others and requires notification within 24 h of clinical diagnosis. Category 2 includes hepatitis infections, lead poisoning, tuberculosis, maternal death, helminth infections and others, and these must be notified within seven days of diagnosis. A smaller list of diseases falls under categories 3 and 4, which require notification within 7 days and 1 month of diagnosis, respectively.^1^ Because these are reported primarily by laboratories, they will not be discussed further in this article.

In 2017, a comparison was carried out between SA’s notifiable disease surveillance system (NDSS) and laboratory disease surveillance. At the time, reporting of NMCs was not mandatory for laboratories. The study demonstrated that there were fewer cases notified by clinicians than were confirmed by laboratory specimens, indicating under-reporting of NMCs by clinicians.^9^ Since then, it has been mandatory for laboratories to report NMCs^10^ as is the standard in many developed countries.^5^ This allows for some conditions that are not reported by clinicians to be identified and reported at the laboratory level. However, should no laboratory confirmation test be requested or carried out, these conditions may still go unreported.

The National Institute for Communicable Diseases (NICD) launched a mobile application in 2018 to replace the paper-based notification system, which had been in use for decades and was last updated in 2017. The application was introduced to provinces in December 2020 and is currently accessed on the personal mobile phones of healthcare workers across the country.^11^ There are limited data available on NMC reporting practices since the development of this application in SA. There is however evidence to suggest that prior to the launch of the application, NMCs were under-reported.^12,13^

It is not uncommon in SA for patients to present to hospital emergency departments (EDs) with a health complaint that is found to be non-urgent. This may add to the patient loads seen in this section of hospitals.^14,15^ The combination of EDs being busy, fast-paced working environments, while also managing patients with primary healthcare-related diseases, could lead to clinicians diagnosing and discharging patients with an NMC without reporting the case. If no laboratory investigations were requested, these diagnoses may go undetected, leading to under-reporting. Cases that are reported by laboratories alone often do not have all the clinical and personal details needed to properly follow up on a reported condition. It is therefore imperative that doctors working in EDs are aware of which conditions require reporting and that these cases are promptly reported.

It is essential to identify hindrances to the reporting of NMCs as early reporting of outbreaks and conditions of concern could improve the public health response. Because EDs are a place where many patients with NMCs first present and are diagnosed, this study aimed to assess ED clinicians’ knowledge, attitudes, and practices regarding the reporting of conditions that are relevant to the ED, and the use of the new mobile application for reporting.

## Research methods and design

A cross-sectional survey of a convenience sample of doctors from three public hospital EDs in Gauteng, SA was carried out from November 2024 to April 2025. These hospitals included Helen Joseph Hospital, Rahima Moosa Mother and Child Hospital, and Tambo Memorial Hospital. It was estimated that approximately 140 doctors are employed or rotating through these EDs at any given time. Interns, community service doctors, medical officers, and registrars were included in the study. Consultants and nurses were excluded because it is rare that they are directly involved in the reporting process in these EDs.

The paper-based questionnaire assessed the knowledge, attitudes, and practices regarding the reporting of NMCs diagnosed in the EDs. It was developed from a similar questionnaire used in a research study carried out in SA in 2018.^16^ We added questions relating to the recently launched notification application by the NICD. Respondents were given an information sheet, which stated that by completing and returning the questionnaire, they consented to participation in the study. The questionnaire also confirmed their right to withdraw at any time and that responses would remain anonymous. Consent was obtained this way because of the low risk of potential harm to respondents.

The questionnaire was divided into four parts. Part one assessed respondents’ knowledge of NMCs with a table listing selected conditions and a tick box for respondents to mark whether the condition is notifiable or not and if notifiable, what the period of notification was (i.e. 24 h or 7 days). Organophosphate poisoning (which is classified under agricultural or stock remedy poisoning by NICD) was assessed as a category 2 NMC in this study because it had only been made a category 1 NMC while this study was being performed, and as such was not widely known yet to be a category 1 NMC.^17^

Part two contained a list of questions in the form of a five-point Likert scale, with options ranging from strongly disagree to strongly agree, assessing attitudes and practices with respect to the reporting of these conditions. Part three included a list of factors that may influence reporting practices; respondents were asked to what degree these influenced their reporting practices. Part four included two separate questions on the preference of reporting method and the ease of use of the NICD application.

The data were then collected and entered manually into a password-protected spreadsheet document. Data were analysed using IBM^®^ Statistical Package for the Social Sciences (SPSS) Statistics version 31. The descriptive statistics present the demographic profile of the study respondents as well as their knowledge, practices and attitudes regarding NMCs. These are reported in counts and percentages for categorical variables, and in mean with standard deviation (s.d.) for numeric variables. The doctors’ knowledge, practices, and attitudes were compared based on their demographic and experience levels using a one-way analysis of variance (ANOVA). Correlations between knowledge, practices, and attitudes were examined using Pearson’s correlation coefficient. A paired sample *t*-test was used to assess for a difference in respondent knowledge accuracy scores between category 1 and category 2 NMCs. In the analyses, *p* < 0.05 indicated statistical significance.

### Ethical considerations

Ethical clearance to conduct this study was obtained from the Human Research Ethics Committee of the University of the Witwatersrand (No. M240826). Participants were given an information sheet, which stated that by completing and handing back the questionnaire, they were providing informed consent to participate in the study. The questionnaire also explained their right to withdraw at any time and that response would remain anonymous. Consent was obtained this way because of the low risk of potential harm to participants.

## Results

### Demographics

A total of 99 responses were received, all of which were used and of which 61% (*n* = 60) were female (Table 1). The age range of respondents was from 25 years to 55 years, with a median age of 30 years. Those 30 years and under comprised 55% (*n* = 54) of respondents, and those 31 years and above made up 46% (*n* = 45). Medical officers made up the largest group of doctors (44%; *n* = 44), with registrars comprising 24% (*n* = 24), interns 19% (*n* = 19), and community service doctors 12% (*n* = 12).

**TABLE 1:** Demographic profile of study respondents.

Demographics	Variable	*n*	%
Age†	Median	30	-
	Minimum	25	-
	Maximum	55	-
Gender	Male	39	39
	Female	60	61
	Total	99	100
Level of training	Intern	19	19
	Community service	12	12
	Registrar	24	24
	Medical officer	44	44
	Total	99	100

† , Interquartile range: 28–33.

### Knowledge of notifiable medical conditions

**FIGURE 1:**
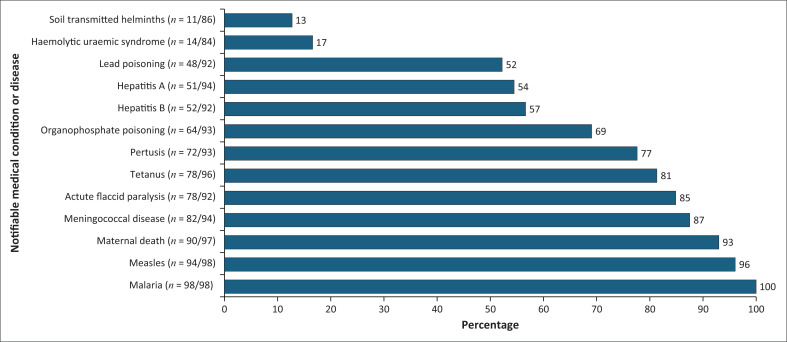
Knowledge accuracy of notifiable medical conditions.

There was no statistically significant difference in knowledge accuracy rates between the respondents at various levels of training; however, respondents’ knowledge accuracy of NMCs varied considerably (Figure 1). Because not every respondent answered each question, the denominators varied accordingly. Certain medical conditions were correctly identified as notifiable by the majority of respondents, namely malaria, measles, acute flaccid paralysis, maternal death, meningococcal disease, pertussis, tetanus and organophosphate poisoning.

Conditions recognised as NMCs by a smaller proportion of the group were hepatitis A and B, helminth infections, lead poisoning and haemolytic uraemic syndrome. Some conditions were reported as notifiable while they were not notifiable, most notably chicken pox (53%; *n* = 49/92), mumps (54%; *n* = 47/87), and cryptococcal infections (52%; *n* = 47/90). The overall respondent knowledge accuracy score corrected for response rates showed a mean of 60.9% (s.d. 11.2).

There was a statistically significant difference in respondents’ knowledge accuracy scores between category 1 and category 2 NMCs, with the mean knowledge accuracy of category 1 and 2 conditions being 72% and 56%, respectively, with a mean difference of 16.1 and s.d. of mean difference of 25.0, *p* < 0.001.

### Attitudes and practices of the notification process

Most respondents (95%; *n* = 90/95) were aware that reporting of an NMC is a legal requirement, and 61% (*n* = 59/97) were aware that laboratory results are not a requirement for reporting. Almost half (49%; *n* = 41/84) of the respondents disagreed or strongly disagreed that they had reported every notifiable condition they had diagnosed, and 32% (*n* = 27/84) reported that they had discharged a patient with uncomplicated measles or malaria without reporting it.

The paper-based notification method was said to be too time-consuming by 64% (*n* = 57/89) of respondents. Many respondents (81%; *n* = 80/99) were aware that there is a notification application available, but only 68% (*n* = 65/96) agreed or strongly agreed that they had used this digital method, and only 17% (*n* = 17/98) said they had received formal training on how to use it.

**FIGURE 2:**
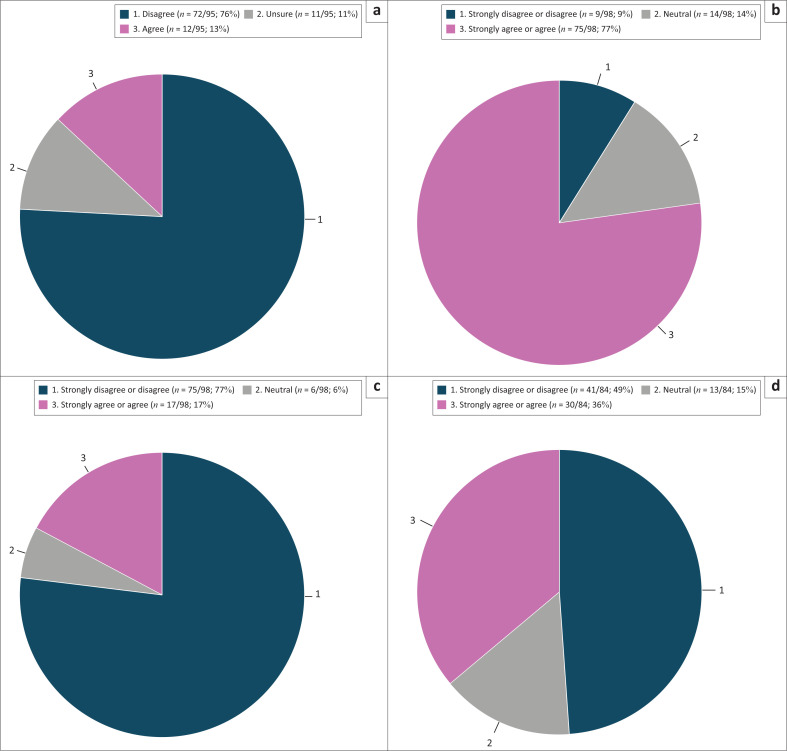
Attitudes and practices of respondents regarding the reporting of notifiable medical conditions: (a) Access to Wi-Fi at work (b) feedback would motivate reporting (c) had formal training on mobile application use (d) reported every notifiable condition diagnosed.

Most of the respondents (76%; *n* = 72/95) said that they do not have access to Wi-Fi at work, and 88% (*n* = 65/74) of all respondents used their own data when reporting a notifiable condition digitally (Figure 2). Despite this, 78% (*n* = 61/78) of respondents preferred the application to the paper-based system, and only 8% (*n* = 8/96) knew what to do with each copy of the completed notification form from the book. Many respondents (77%; *n* = 75/98) agreed or strongly agreed that feedback on their notifications would motivate them to report more consistently.

**FIGURE 3:**
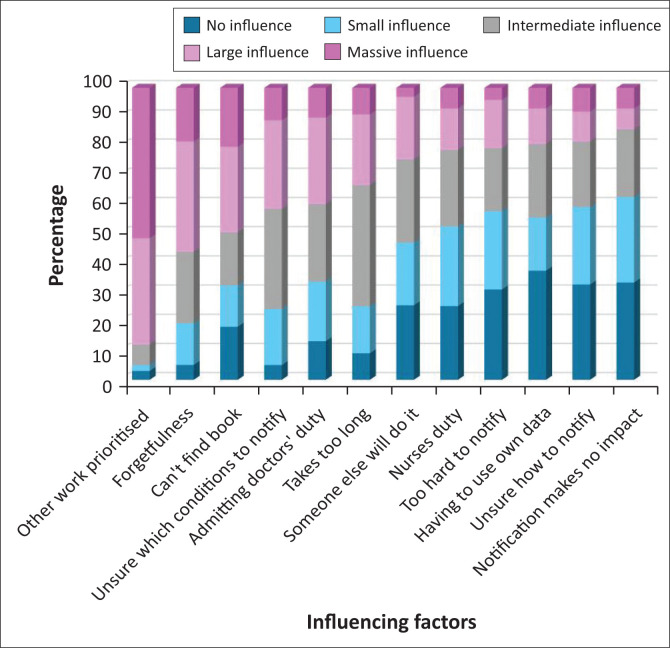
Factors negatively influencing the reporting of notifiable conditions.

The greatest reported barriers to respondents reporting an NMC were other work taking priority, forgetfulness and difficulty locating the notification booklet (Figure 3). Having to use one’s own cellular data to report conditions had a small impact on the notification practices of respondents. Factors that had less impact on notification practices included not knowing how to report, the process being too difficult, the feeling that reporting does not make a difference and the belief that reporting is the duty of other staff members including nursing staff.

Of the respondents who had used the NICD notification application, 64% (*n* = 39/61) found it easy or very easy to use with only 7% (*n* = 4/61) finding its use difficult. The application was by far the most preferred method to report conditions (79%; *n* = 77/98) versus 20% (*n* = 20/98) preferring the booklet.

## Discussion

This study included doctors at various experience and training levels to reflect the diversity of staff in EDs involved in the notification process. Knowledge gaps were demonstrated with category 1 conditions having had better knowledge accuracy rates than category 2 conditions, and some non-notifiable conditions inaccurately identified as notifiable. The results also revealed under-reporting of NMCs. Despite widespread knowledge of the mobile application, there remained low levels of use reported as well as very low levels of formal training received. The application still appeared to be a preferred method for reporting despite there being minimal access to Wi-Fi at places of work. Workload was shown to be a major contributing factor to the under-reporting of NMCs with feedback on cases identified as a possible motivating factor.

While consultants and nurses were excluded from this study, it is worth noting that they may report NMCs. Although it may not be common practice for them to do so, their knowledge of which conditions are notifiable is still of importance in helping to identify cases that require reporting. The outcomes of the knowledge component of the study could be partially explained by the frequency with which certain conditions are seen and therefore reported. Malaria, for example, had a 100% knowledge accuracy rate and is one of the most notified category 1 conditions.^18^ The knowledge accuracy scores for category 1 NMCs were better than those of category 2 conditions, indicating that ED staff had better knowledge of diseases that require more urgent notification than diseases that allow for a longer notification period. These conditions are likely better remembered because of their public health significance and the severity of disease they have the potential to cause.

Interestingly, the knowledge accuracy for organophosphate poisoning, a category 1 NMC that forms part of agricultural and stock remedy poisoning and is a commonly seen condition in South African hospitals,^19^ was much lower than that of malaria. This could be explained by the fact that up until recently, it was a category 2 notifiable NMC in SA, which required notification within seven days of diagnosis.^17,20^ As a result, ED staff were often not the individuals who reported this condition and could therefore have been less aware of it being notifiable within 24 h.

Additionally, it is not currently common practice to confirm the diagnosis of organophosphate poisoning with laboratory results, and the condition is often treated based on history and clinical examination alone. Since only 61% of respondents were aware that laboratory results are not always required to report a condition, it may be that certain conditions are not notified from the ED due to the inaccurate belief that there needs to be laboratory confirmation first. This points to a lack of training regarding NMCs and a need for more educational interventions on this topic.

Hepatitis A and B, helminth infections, lead poisoning and haemolytic uraemic syndrome had relatively low knowledge accuracy scores, displaying an important knowledge gap and the need for improved training in this area. These conditions do require laboratory confirmation to make the diagnosis, and since these patients often get admitted or followed up elsewhere, ED staff frequently do not see these laboratory results to confirm the diagnosis and report it. It remains important that doctors are aware that these conditions are notifiable should they make the diagnosis.

The conditions inaccurately said to be notifiable again highlight a knowledge gap. It could also demonstrate that although one might suspect a notifiable condition in practice, they are not always being reported because if one were to look for these conditions on the notification application to report them, they would not be listed. This is supported by the finding that 49% of respondents admitted to not always reporting every notifiable condition they had diagnosed, despite 95% of respondents being aware that it is a legal requirement to do so.

This study shows that not all notifiable conditions identified in these EDs are being reported, as has been found in other SA studies that demonstrated under-reporting of NMCs.^9,12,13^ This is a public health concern because for many patients with these conditions, the initial – and often only – point of contact with a healthcare facility is in the ED, and a delay or absence of reporting could impact timely identification and response to prevent the spread of the disease.

There was widespread knowledge of the notification application, but still, many respondents had not used it as a means of reporting these conditions. This could be because of the lack of formal training in its use (which only 17% of respondents reported having received), leading to a lack of motivation or discomfort in trying something new while on a busy ED shift. Given the demanding and continuous nature of work in EDs, staff may prefer to continue with familiar and established practices rather than trying to learn a new skill in an already pressured environment. This emphasises the importance of formal training and motivation when trying to implement these new practices. EDs also often have a high turnover of staff, including sessional or part-time staff members, which may be a contributing factor to the lack of knowledge and training.

Although the use of the application was not widespread among respondents, it still appeared to be the preferred method of reporting when compared to the paper-based system, despite most respondents not having access to Wi-Fi at their places of work. Although the question of reporting method preferences specifically referred to the NICD notification application, respondents may have answered the question more generally in that they preferred any application over the paper-based method because 78% of respondents preferred the application, but only 68% reported having used it.

Interestingly, having to use own cellular data did not seem to have a major impact on notification practices. This could be explained by the fact that it is, and has been, common practice for doctors in public hospitals to use their personal phones and mobile data to look up blood results and perform other work-related tasks.^21^ Despite this, improving access to Wi-Fi at hospitals may increase rates of use of the application and aid in faster reporting and response to these conditions.

According to respondents, general workload had the biggest negative effect on the reporting of notifiable conditions. Workload was also found to be a contributing factor to under-reporting in the study by Benson.^16^ This represents more of a system-wide hindrance to reporting because of increased patient load and chronic understaffing.^14,22^ These matters place notifying these conditions further down on the priority list of tasks, which could impact timely identification of outbreaks for infectious diseases and recognition of patterns of concern for non-infectious conditions. A lack of formal training on the use of the mobile application may also serve as a barrier to reporting. The majority of respondents felt that receiving feedback from NICD about notified conditions would motivate them and possibly improve reporting rates among respondents. This is something to consider building into the reporting application in the form of updates on reported cases and local disease spread to make notifiers aware that their actions are acted upon and do make a difference.

### Limitations and strengths

The study’s limitation is that a convenience sample was used, leading to a small sample size. Not all questions were answered by all respondents, further narrowing the sample size for some of the questions. This was a paper-based, tick-box style questionnaire that required manual capturing of data; however, every effort was made to minimise human error in this process. Because the study was a self-reported questionnaire, there could be a degree of social desirability bias despite the questionnaire being anonymous. Lastly, the exclusion of nurses and consultants from the study may be viewed as a limitation because they are also able to report conditions or remind others to do so.

The study’s strength is that it provides insight into an important aspect of the healthcare system and specifically public health and offers some considerations for when digitisation of a disease reporting system is planned here and elsewhere in Africa. With the recent digitisation of the NMC reporting system, there is a need to assess its use and favourability as well as factors contributing to its underuse.

## Conclusion

This study revealed that there is a knowledge gap in which conditions are notifiable among doctors working in EDs, more so with category 2 NMCs. Respondents acknowledged under-reporting of NMCs, with the main contributing factor being increased workload. The new mobile application for reporting NMCs appears to be a preferred method of reporting in comparison to the paper-based system, indicating an encouraging step forward in improving the timely notification of these conditions. There does however appear to be a lack of formal training in the use of the application, which could hinder successful usage. Overall under-reporting of NMCs among ED doctors is still a concern that requires ongoing staff reminders and training.

## References

[1] National Institute for Communicable Disease. NICD Notifiable medical conditions brochure [homepage on the Internet]. 2025 [cited 2025 Dec 22]. Available from: https://www.nicd.ac.za/wp-content/uploads/2025/07/NMC-Brochure_Updated_2025.pdf

[2] Badker R, Miller K, Pardee C, et al. Challenges in reported COVID-19 data: Best practices and recommendations for future epidemics. BMJ Glob Health. 2021;6(5):1–8. 10.1136/bmjgh-2021-005542 PMC8103560 33958393

[3] Rother HA. Improving poisoning diagnosis and surveillance of street pesticides. S Afr Med J. 2012;102(6):485–488. 10.7196/SAMJ.5838 22668944

[4] National Institute for Communicable Diseases. NICD Notifiable medical conditions overview. 2023 [cited 2025 Dec 21]. Available from: https://www.nicd.ac.za/nmc-overview/overview/

[5] Girdler-Brown BV. Evaluation of the notifiable diseases surveillance system in South Africa. Int J Infect Dis. 2017;59:139–140. 10.1016/j.ijid.2017.03.022 28528937

[6] Doyle TJ, Glynn MK, Groseclose SL. Completeness of notifiable infectious disease reporting in the United States: An analytical literature review. Am J Epidemiol. 2002;155(9):866–874. 10.1093/aje/155.9.866 11978592

[7] Roush S, Birkhead G, Koo D, Cobb A, Fleming D. Mandatory reporting of diseases and conditions by health care professionals and laboratories. JAMA. 1999;282(2):164–170. 10.1001/jama.282.2.164 10411198

[8] Jansson A, Arneborn M, Ekdahl K. Sensitivity of the Swedish statutory surveillance system for communicable diseases 1998–2002, assessed by the capture-recapture method. Epidemiol Infect. 2005;133(3):401–407. 10.1017/S0950268804003632 15962546 PMC2870263

[9] Benson FG, Musekiwa A, Blumberg L, Rispel LC. Comparing laboratory surveillance with the notifiable diseases surveillance system in South Africa. Int J Infect Dis. 2017;59:141–147. 10.1016/j.ijid.2017.03.007 28532981

[10] South Africa. Department of Health. National Health Act, 2003 (Act no. 61 of 2003) Regulations relating to the surveillance and the control of notifiable medical conditions [homepage on the Internet]. Government Gazette No 41330:1434. 2017 [cited 2024 Jan 24]. Available from: https://www.gov.za/documents/notices/national-health-act-regulations-surveilance-and-control-notifiable-medical

[11] NICD Division of Public Health Surveillance and Response. Uptake of the improved NMC App, South Africa. Communicable Diseases Communiqué [serial online]. 2022 [cited 2024 Mar 15];21(1):2. Available from: https://www.nicd.ac.za/wp-content/uploads/2022/01/NICD-Monthly-Communique-January.pdf

[12] Nkgudi B, Robertson K, Volmink J, Mayosi B. Notification of rheumatic fever in South Africa – Evidence for underreporting by health care professionals and administrators. S Afr Med J [serial online]. 2006 [cited 2025 Nov 11];95(3):206–208. Available from: https://journals.co.za/doi/epdf/10.10520/EJC68680 16607429

[13] Mathatha ED, Manamela JM, Musekiwa A, Prabdial-Sing N. Exploring the knowledge, attitudes and practices (KAP) of health care professionals on viral hepatitis notification in Gauteng, South Africa, 2015. Arch Public Health. 2018;76:75. 10.1186/s13690-018-0319-8 30555693 PMC6282396

[14] Thaba T, Madela-Mntla E, Ramochele M, Nzaumvila D. Reasons for avoiding their local clinics among non-emergency patients at accident and emergency department, Pretoria, South Africa. Open Public Health J. 2022;15(1):1–8. 10.2174/18749445-v15-e221129-2022-55

[15] Hodkinson PW, Wallis LA. Cross-sectional survey of patients presenting to a South African urban emergency centre. Emerg Med J. 2009;26(9):635–640. 10.1136/emj.2008.063362 19700577

[16] Benson FG, Levin J, Rispel LC. Health care providers’ compliance with the notifiable diseases surveillance system in South Africa. PLoS One. 2018;13(4):1–16. 10.1371/journal.pone.0195194 PMC5891014 29630627

[17] South Africa. Department of Health. National Health Act, 2003 (Act no. 61 of 2003) Regulations relating to the surveillance and the control of notifiable medical conditions: amendment [homepage on the Internet]. Government Gazette No 52391:6064. 2025 [cited 2025 Nov 15]. Available from: https://www.gov.za/documents/notices/national-health-act-regulations-surveillance-and-control-notifiable-medical-2

[18] The National Institute for Communicable Diseases. Notifiable medical conditions surveillance system report for August 2025 [homepage on the Internet]. 2025 [cited 2025 Nov 15]. Available from: https://www.nicd.ac.za/wp-content/uploads/2025/11/NMCSS-August-2025.pdf

[19] Omar S, Bahemia IA, Toerien L, San Pedro KM, Khan AB. A retrospective comparison of the burden of organophosphate poisoning to an Intensive Care Unit in Soweto over two separate periods. Afr J Emerg Med. 2021;11(1):118–122. 10.1016/j.afjem.2020.09.007 33680732 PMC7910160

[20] National Institute for Communicable Disease. Notifiable Medical Conditions (NMC) case definitions flipchart: Category 1 conditions [homepage on the Internet]. 2025 [cited 2025 Nov 01]. Available from: https://www.nicd.ac.za/wp-content/uploads/2025/08/NMC_category-1-case-definitions_Flipchart_July-2025.pdf

[21] Xu Y, Francis Z, Saleem K, et al. Usage of smart devices amongst medical practitioners in Universitas Academic Hospital. S Afr Fam Pract. 2020;62(1):1–7. 10.4102/safp.v62i1.5029 PMC8378102 32148052

[22] Rauf W, Blitz JJ, Geyser MM, Rauf A. Quality improvement cycles that reduced waiting times at Tshwane District Hospital Emergency Department. S Afr Fam Pract. 2008;50(6)43a–43e. 10.1080/20786204.2008.10873781

